# Everyday Prospective Memory and Executive Function Deficits Associated with Exposure to Second-Hand Smoke

**DOI:** 10.1155/2013/160486

**Published:** 2012-09-18

**Authors:** Thomas M. Heffernan, Terence S. O'Neill

**Affiliations:** Collaboration for Drug and Alcohol Research (CDAR), Division of Psychology, Department of Psychology, Northumbria University, Newcastle upon Tyne NE1 8ST, UK

## Abstract

This study explored whether exposure to second-hand smoke (SHS) has a detrimental impact upon everyday memory in two groups of non-smokers; one which reported regular exposure to SHS and one that reported never having been exposed to SHS. Thirty-four non-smokers who reported having been regularly exposed to SHS (SHS group) and 34 non-smokers who reported never having been exposed to SHS (non-SHS group) were compared on self-reports of prospective memory (PM: remembering future intentions and/or activities) and executive function (EF: those processes involved in attention, multitasking and decision-making). The Prospective and Retrospective Memory Questionnaire (PRMQ) assessed everyday PM lapses; the Executive Function Questionnaire (EFQ) assessed self-reported problems in EF; a drug-use questionnaire and a mood questionnaire were also administered. Two univariate ANCOVAs were applied to the PM and EF data, controlling for between-group differences in age, weekly alcohol use, anxiety and depression scores, and self-reported retrospective memory scores. The SHS group reported significantly more lapses on the PRMQ and more deficits on the EFQ than the non-SHS group. These findings provide new insights into PM and EF deficits associated with prolonged exposure to SHS in a group of non-smokers. Possible explanations and suggestions for future research are also considered.

## 1. Introduction

Second-hand smoke (SHS) refers to a situation where one person inhales another person's smoke either by exposure to side stream smoke (smoke emitted from the end of a cigarette, pipe, or cigar) or mainstream smoke (the smoke that is exhaled by the smoker directly). Previous research has suggested that exposure to second-hand smoke (SHS) not only has a detrimental effect upon health, including cardiovascular disease [1, 2], but also is associated with poorer cognitive performance in children, adolescents, and adults (3–6). For example, children exposed to SHS show reduced vocabulary and reasoning abilities [3], as well as more general cognitive and intellectual deficits [4]. In addition, recent work has shown a strong relationship between exposure to SHS and impairments in reading, mathematics, and visuospatial skills in children and adolescents [5] and poorer cognitive function in adults [6, 7]. In the first of these adult studies [6], participants included in the study had no history of smoking or using any tobacco product, and had no history of cardiovascular disease or dementia. Based on their self-reported long-term exposure to SHS tobacco smoke (having lived with a smoker for 3 decades) the study found that those exposed to SHS were about 30% more likely to develop dementia over a period of six years when compared with those who reported never having been exposed. In the second of these adults studies [7] participants who had no history of smoking any tobacco product were measured on exposure to SHS using cotinine biomarker assays. In a cross-sectional design, participants exposed to different levels of SHS were compared on cognitive measures including processing speed and executive function. The findings from the study revealed that exposure to increasingly higher levels of SHS corresponded with greater deficits in cognitive function. From this research it can be concluded that exposure to SHS in never-smoked groups equates to deficits in cognitive function. These deficits have been linked to risk factors, such as cardiovascular disease, associated with SHS in the past [8, 9]. These data suggest that exposure to SHS may be causally associated to impairments in a range of cognitive processes. However, what is not clear is whether exposure to second-hand smoke is associated with impairments in everyday cognition, of which *prospective memory* and *executive function* are two good examples. 

 Prospective memory (PM) is an important aspect of day-to-day memory function and refers to the process of remembering to do things at some future point in time [10]. For example, remembering to attend an appointment at a clinic, remembering to carry out a task such as paying a bill on time or remembering to take ones medication on time are all examples of PM. Executive function (EF) is an umbrella term that is used to describe a collection of processes making up the central executive component of the working memory model [11, 12] and includes planning, task coordination, impulse control, and attention. PM is thought to be critical to independent living [13] and a compromised EF is likely to lead to confusion, poor planning, and other executive problems on everyday tasks, so developing a greater understanding of how both these sets of processes might be affected by exposure to potentially harmful substances, such as SHS exposure. 

There is good evidence that performance on PM tasks relies on prefrontal systems in the brain and on the integrity of related EF [14–18]. Frontally mediated EF is believed to play key roles in a range of processes, including planning a task, monitoring one's environment, the inhibition of extraneous responses, and cognitive flexibility [19–21]. For example, research has shown that when high demands are placed on EF (using a dual-task paradigm) executive processes (measured using the Tower of London task, the Stroop task, and the Wisconsin Card Sorting Task) predicted performance on the more complex dual-task PM paradigms, but not on simple (single) PM task paradigms [20]. This supports the notion that frontal/executive functions are intimately related to PM performance. Furthermore, evidence from brain-imaging studies also highlights strong links between PM and EF [19, 22]. Given that prolonged exposure to SHS is linked to deficits in EF [7] and based on the links between PM and EF discussed here, it is feasible to hypothesise that prolonged exposure to SHS in a never-smoked group may lead to impairments in both EF and PM in the same cohort, when compared with a group who have never smoked and whom have not been exposed to SHS. The issue of what physical harm exposure to SHS has upon the individual is gaining international interest [23], yet despite this there is very little in the way of systematic research into what impact SHS exposure has upon everyday cognition in nonsmoking adults. The current study seeks to address this by comparing two groups of nonsmokers (individuals who had never smoked): one of which reported regular exposure to SHS and one that reported never having been exposed to SHS, upon self-reports of PM and EF. Since other drug use can independently impede PM performance [24, 25] and given that variations in mood can negatively affect cognition [26, 27], these were measured and included in the main analysis as covariates. Finally, since retrospective memory (RM) deficits have been found in those exposed to SHS and given the RM is related to both PM and EF [19], self-reported RM was also gauged and included as a covariate. 

## 2. Method

### 2.1. Participants

An original sample of 150 individuals attending a university in the North East of England was recruited. From this original sample, 82 were omitted on the basis that they reported using an illegal substance (e.g., cannabis, ecstasy), were or had been smokers, and/or were heavy drinkers or had drunk any alcohol within the last 48 hours, and/or had reported a psychiatric illness (e.g., depression, substance dependence). Of the remaining 68 participants, 34 were non-smokers who reported that they had been regularly exposed to second-hand smoke (SHS) either in a home or social situation (the SHS group) and 34 were nonsmokers who had reported never having been exposed to SHS (the non-SHS group). In the SHS group (29 females, 5 males; mean age = 20.2 years, S.D.: 2.73) 70% of them reported that their exposure to SHS came from the home which they shared with smokers and 30% reported that their exposure came from a social setting within which they sat with smokers in a partially confined “smoking hut/area” outside a pub/restaurant/bar. This group reported that they were exposed to SHS for an average of 13.8 hours per week, S.D.: 16.9, and had been exposed in this way for an average of 6.14 years, S.D.: 6.04. They also reported that they drank on average 10.2 units of alcohol per week, S.D.: 7.60, had been drinking for an average of 4.00 years, S.D.: 2.39, and had not drunk for an average of 103 hours, S.D.: 94. The non-SHS group (27 females, 7 males; mean age = 19.4 years, S.D.: 0.86) reported that they had never been directly exposed to SHS either at home, at work, at university, or within a social situation; they reported that they drank on average 8.47 units of alcohol per week, S.D.: 6.08, had been drinking for an average of 3.88 years, S.D.: 1.98, and had not drank for an average of 117 hours, S.D.: 108. As stated previously all 68 participants reported they did not use any illegal substance in addition to alcohol. 

## 3. Measures and Procedure

 Participants completed a series of brief questionnaires. A drug-use questionnaire was completed in which smoking and other drug use were assessed using a modified version of a Recreational Drug Use Questionnaire (RDUQ) used in previous research [24, 25]. This measured their smoking status; the number of hours exposed per week to SHS, the situation in which they were exposed and the number of years exposed to SHS (relating to the SHS group); the number of alcohol units consumed per week, length of alcohol use in years and when they last drank alcohol in hours. Similar questions were asked in relation to other drug use (e.g., cannabis, ecstasy). There were also “nonuse” options for all these drugs. Demographics (age, gender), whether they had previously suffered from/or were currently suffering from a substance dependence disorder, clinical amnesia, or some other psychiatric condition, were also measured on the questionnaire. 

 As previous research has indicated that there may be an association between depression and cognitive failures [26, 27], all participants completed the Hospital Anxiety and Depression Scale (HADS) [28] which is a 14-item standardised self-report questionnaire. Seven items measured generalised anxiety symptoms and 7 measured generalised depressive symptoms. Separate overall scores were obtained for the anxiety and depression constructs, each ranging from between 0 to 21, with a higher score indicating more severe symptoms. The HADS has been shown to be a valid and reliable measure of mood in nonclinical samples [29]. 

Prospective memory was assessed using the PM scale from the Prospective and Retrospective Memory Questionnaire (PRMQ) which is a self-report measure developed by previous authors [30]. The retrospective memory (RM) subscale of the PRMQ was also calculated since RM is related to both PM and EF. The PRMQ shows high internal consistency, with the reliability on Cronbach's alpha being 0.89. The PRMQ assesses self-reported prospective and retrospective memory slips in everyday life.  Table 1 contains the full list of PM and RM questions contained in the PRMQ. The participant rated how often they experienced such failures on a 5-point scale from “very often” (5) to “never” (1) by circling the response that best reflects their memory ability. A mean score for PM slips/failures was calculated, along with a mean score for RM slips/failures, in both cases with a higher score indicating more memory slips/failures. 

 Executive function was measured using an Executive Function Questionnaire (EFQ) devised and validated by previous research [31]. The questionnaire is comprised of a series of questions designed to estimate deficits in the main components of executive function—including attentional difficulties, problems in concentration, one's ability of multitask, perseverance on a task, and impulse control. The EFQ shows high internal consistency, with the reliability on Cronbach's alpha being 0.78. For each item, participants responded by circling one response from a four-point scale (1) no problems experienced; (2) a few problems experienced; (3) more than a few problems experienced; (4) a great many problems experienced.  Table 2 contains the full list of executive questions contained in the EFQ. The total scale score was computed by summing the responses to the six items and this total score was intended to reflect the participant's overall experience of executive problems rather than any specific aspect thereof, with a higher score indicating more executive deficits experienced. 

The research received ethical approval from the School of Life Sciences Ethics Committee at Northumbria University. Participants were recruited on a voluntary basis and tested individually in a controlled laboratory situation. The PRMQ was administered first, followed by the EFQ, HADS, and then the personal characteristics/drug-use questionnaire. After completing the study participants were thanked for their cooperation and fully debriefed. 

## 4. Results and Discussion

Chi-square analysis revealed no significant difference in the number of males and females between the SHS and non-SHS groups (*χ* ^2^(1) = 0.40, *P* = 0.52). In order to observe what independent impact each of the covariates had upon prospective memory (PM) and executive function (EF), as well as what impact exposure to SHS has upon PM and EF after controlling for these covariates, two univariate ANCOVAS were applied to the PM and EF data (controlling for age, weekly alcohol use, anxiety and depression scores, and self-reported retrospective memory (RM) scores). The first ANCOVA revealed no significant independent impact of age upon PM *F* (1, 62) = 0.79, *P* = .37, no significant independent impact of weekly alcohol use upon PM *F* (1, 62) = 2.17, *P* = .14, no significant independent impact of HADS anxiety upon PM *F* (1, 62) = 0.29, *P* = .59, no significant independent impact of HADS depression upon PM *F* (1, 62) = 0.24, *P* = .95, but RM did have a significant impact upon PM *F* (1, 62) = 73.8, *P* < .001. After controlling for variations in these covariates a significant impact of SHS upon PM remained *F* (1, 62) = 4.33, *P* < .05. Inspection of the means showed that the SHS group reported more PM errors than the non-SHS group (see Table 3). The second ANCOVA revealed no significant independent impact of age upon EF *F* (1, 62) = 0.00, *P* = .98, no significant independent impact of weekly alcohol use upon EF *F* (1, 62) = 1.59, *P* = .21; however, there was a significant independent impact of HADS anxiety upon EF *F* (1, 62) = 8.80, *P* < .01, and a significant independent impact of HADS depression upon EF *F* (1, 62) = 7.66, *P* < .01, as well as a significant independent impact of RM upon EF *F* (1, 62) = 4.33, *P* < .05. After controlling for variations in these covariates a significant impact of SHS upon EF remained *F* (1, 62) = 4.32, *P* < .05. Inspection of the means showed that the SHS group reported more EF deficits than the non-SHS group (see Table 3). A Pearson Product Moment correlation revealed a significant positive correlation between scores on the EF and PM measures* r* (68) = .215, *P* < .05, indicating more failures reported on the EF measures corresponded with greater memory lapses on the PM measure. 

The main finding from this study was that both increased frequency of self-reported PM lapses and self-reported deficits in EF were associated with exposure to second-hand smoking in the SHS group when compared with the non-SHS group. Thus, those participants who had never smoked but who reported being regularly exposed to SHS in confined spaces for prolonged periods of time on several occasions per week and over several years showed significantly more forgetting on everyday PM tasks (such as forgetting future activities one had planned to do) as well as greater deficits in EF (such failures in attention, planning, and multitasking), when compared with a group of never smokers who had not been exposed to SHS. To our knowledge this is the first analysis to observe a relationship between SHS exposure in a never-smoked group and both PM and EF deficits within the same cohort of participants. We controlled for a wide range of covariables that are potential confounders in cognitive research. Having reduced PM capabilities can result in poorer performance on everyday tasks, such as remembering meetings, chores one has to perform, and so forth, and having a compromised EF can only add to these problems. It is important therefore to observe whether the findings here are replicable under a variety of tasks that tap into PM and EF. Given that this was a relatively young cohort (with 97% of the SHS exposed group being under the age of 25 years (100% in the case of the non-SHS group)), the findings suggest that putative cognitive deficits as a result of prolonged exposure to SHS can start to occur after a relatively short period of time (the mean SHS exposure time in this study was just over 6 years) even in young people. 

As suggested earlier, these may be important findings, since only a handful of studies to date have observed cognitive and intellectual deficits associated with prolonged exposure to SHS in children [3, 4], as well as observing an association between SHS exposure and deficits in the neurocognitive function of adolescent and adult populations [5–7], but none of these previous studies have assessed cognitive function directly, nor have they done so in relation to everyday remembering. Given that PM and EF are seen as essential to independent living [13, 19], exploring the relationship between exposure to SHS and deficits in these cognitive domains may be of paramount importance. A particular strength of this study was the number of “controls” adopted; that is, anyone who reported using an illegal drug (such as ecstasy or cannabis), who drank heavily or had drunk any alcohol within the past 48 hours, or reported suffering from a clinical psychiatric condition as excluded from the study. The findings were also observed after controlling for between-group variations on age, gender, weekly alcohol use, mood (anxiety and depression scores), and self-reported retrospective memory scores. Although the present study found a relationship between exposure to SHS, reduced PM performance, and deficits in EF which were more profound in a never-smoked group who were exposed to SHS, the precise nature of this relationship needs further exploration in future research. Since there is evidence that preexisting deficits in EF are associated with more “risky” behaviour, including a greater risk of drug taking and more risky sexual activity, [32]. It is feasible that premorbid deficits in EF has resulted in greater exposure to SHS which, in turn, has led to the impairments in everyday PM found here. Those in the non-SHS exposure group may simply have had more proficient EF, opted not to engage in “risky behavior” (i.e. not expose themselves to SHS) and therefore have a more intact everyday PM as a result. This does not rule out the possibility that prolonged exposure to SHS results in decrements in both EF and PM. Future research should test these competing hypotheses by employing a longitudinal study comparing all neversmokers on EF scores before they became exposed to SHS and then comparing these with postexposure PM scores before any firm causality can be established. 

The current study used self-report measures of both PM and EF. Whilst self-reports of both PM and EF have been useful in uncovering deficits in these domains in a range of drug users in the past, including excessive alcohol users [24] and ecstasy/cannabis polydrug users [25], it would be advantageous to confirm such findings using objective measures of PM and EF. Using objective measures alongside self-reports can provide convergent evidence of PM and EF deficits associated with exposure to SHS, which can only act to bolster the argument that prolonged exposure to tobacco smoke leads to cognitive impairments, for example, the CAMPROMPT which assesses time-and-event based PM [33] and the Reverse Digit Span as an objective measure of EF [11]. The reliance on self-reports of memory lapses in a group who may already have compromised memory problems due to prolonged SHS exposure (the SHS group) raises the possibility of a “memory paradox,” in which participants with faulty memories may inaccurately recall their memory failures. However, given that this is not a highly publicised area within the public domain (due to the scarcity of research in this field) it is unlikely that the SHS group would have a heightened awareness of the everyday memory problems associated with exposure to SHS. Indeed, several studies have shown that participants with a range of pathologies are more likely to* underestimate* their memory deficits [34, 35]. Further investigations, including the use of objective measures of PM and EF, as well as longitudinal studies that plot the decline in everyday memory associated with a greater length of time exposed to SHS, are also needed. One important area for further work would be to elucidate the relationship between biological mechanisms, SHS exposure, and cognitive deficits (such as the ones observed in this study). For example, exposure to SHS in neversmokers has been found to lead to a range of cardiovascular diseases similar to those observed in active smokers [1, 2], which may in turn lead to an increased risk of cognitive impairment in adults [7]. Future work should consider measuring health indices alongside PM and EF in never smokers exposed to SHS in order to test whether it is SHS-exposure-related cardiovascular disease that may account for the deficits in PM and EF found here. Given that some recent work from animal studies suggests that prolonged exposure to the toxic mixtures emitted in tobacco smoke, such as the tobacco-specific procarcinogen 4-methylnitrosamino-1-(3-pyridyl)-1-butanone (NNK), causes neuronal damage in the brain [36], this too could offer potential for further work in order to observe whether direct neuronal damage from SHS leads to compromised cognitive function. Further work in this area should also assess a range of other potential cohort differences, such as socioeconomic status and lifestyle variables—since these too might be linked with “risky behavior.” Finally, research could also be extended to observe what impact exposure to SHS has upon the health and cognition of children, an area that is fast becoming a major public health concern, one that is highlighted by the recent World Health Organisation reports on the global epidemic of smoking, including exposure to SHS [23].

## 5. Conclusion

Not only do the findings of this study confirm previous research indicating a range of memory deficits associated with SHS exposure in a never-smoked group, but also they have demonstrated both PM and EF deficits within the same cohort of never smokers who have been exposed to SHS, which are not found in a group of never smokers not exposed to SHS. We hope that the findings uncovered here act to improve knowledge about the wider effects of SHS exposure, specifically in relation to the everyday cognitive consequences found here. It is a further hope that the results obtained here can be of help in campaigns that raise awareness of the dangers of SHS exposure beyond the already established health consequences.

## Figures and Tables

**Table 1:** Self-reported memory slips for prospective memory items (questions: 1, 3, 5, 7, 10, 12, 14, 16) and retrospective memory items (questions: 2, 4, 6, 8, 9, 11, 13, 15).

(1) Do you decide to do something in a few minutes' time and then forget to do it?				
Very often	Quite often	Sometimes	Rarely	Never
(2) Do you fail to recognize a place you have visited before?				
Very often	Quite often	Sometimes	Rarely	Never
(3) Do you fail to do something you were supposed to do a few minutes later even though it is there in front of you, like taking a pill or turning off the kettle?				
Very often	Quite often	Sometimes	Rarely	Never
(4) Do you forget something that you were told a few minutes before?				
Very often	Quite often	Sometimes	Rarely	Never
(5) Do you forget appointments if you are not prompted by someone else or by a reminder such as a calendar or diary?				
Very often	Quite often	Sometimes	Rarely	Never
(6) Do you fail to recognize a character in a radio or television show from scene to scene?				
Very often	Quite often	Sometimes	Rarely	Never
(7) Do you forget to buy something you planned to buy, like a birthday card, even when you see the shop?				
Very often	Quite often	Sometimes	Rarely	Never
(8) Do you fail to recall things that have happened to you in the last few days?				
Very often	Quite often	Sometimes	Rarely	Never
(9) Do you repeat the same story to the same person on different occasions?				
Very often	Quite often	Sometimes	Rarely	Never
(10) Do you intend to take something with you, before leaving a room or going out, but minutes later leave it behind, even though it is there in front of you?				
Very often	Quite often	Sometimes	Rarely	Never
(11) Do you mislay something that you have just put down, like a magazine or glasses?				
Very often	Quite often	Sometimes	Rarely	Never
(12) Do you fail to mention or give something to a visitor that you were asked to pass on?				
Very often	Quite often	Sometimes	Rarely	Never
(13) Do you look at something without realising you have seen it moments before?				
Very often	Quite often	Sometimes	Rarely	Never
(14) If you tried to contact a friend or relative who was out, would you forget to try again later?				
Very often	Quite often	Sometimes	Rarely	Never
(15) Do you forget what you watched on television the previous day?				
Very often	Quite often	Sometimes	Rarely	Never
(16) Do you forget to tell someone something you had meant to mention a few minutes ago?				
Very often	Quite often	Sometimes	Rarely	Never

**Table 2:** The executive items from the executive function questionnaire.

(1) Do you find it difficult to keep your attention on a particular task?	
(2) Do you find yourself having problems concentrating on a task?	
(3) Do you have difficulty carrying out more than one task at a time?	
(4) Do you tend to “lose” your train of thoughts?	
(5) Do you have difficulty seeing through something that you have started?	
(6) Do you find yourself acting on “impulse”?	

**Table 3:** Adjusted mean scores on the self-reported PM lapses and EF deficits.

	Self-reports	
	PM lapses	EF deficits
SHS group	2.84 (0.54)	14.0 (3.69)
Non-SHS group	2.36 (0.47)	11.3 (2.38)

Note. Standard errors are given in brackets.
